# Pseudo‐Hypoaldosteronism Type 2 due to New Variants of KLHL3 Gene Diagnosed in an Adult Woman With Very High Sensitivity to Hydrochlorothiazide

**DOI:** 10.1002/ccr3.70897

**Published:** 2025-09-15

**Authors:** Massimo Giusti, Monica Repetto, Carmela Caputo

**Affiliations:** ^1^ Endocrine Unit Centro Clinico Diagnostico Priamar Savona Italy; ^2^ Dipartimento di Medicina Interna Università di Genova Genoa Italy; ^3^ Nephrology and Dialysis Unit San Paolo Hospital Savona Italy; ^4^ Kidney Transplant Center, Pediatric Nephrology and Dialysis Unit Santobono Hospital Naples Italy

**Keywords:** hyperkalemia, hypertension, KLHL3 gene variants, pseudo‐hypoaldosteronism type 2, sensitivity to hydrochlorothiazide

## Abstract

We report a case of pseudo‐hypoaldosteronism type 2 (PHA II) in a hypertensive 55‐year‐old woman who carried new variants of the KLHL3 gene. Hypersensitivity to hydrochlorothiazide was noted. Low dosages of hydrochlorothiazide were needed to restore potassium levels. PHA II must be excluded in adult hypertensive subjects with unexplained hyperkalemia.

## 1 Introduction

Pseudo‐hypoaldosteronism type 2 (PHA II), also known as Gordon syndrome, is a rare disorder associated with positive Na+ balance and renal K+ retention, causing hypertension, hyperkalemia, and metabolic acidosis with a normal glomerular filtration rate. Renin and aldosterone levels are variable. PHA II is caused by variants of several genes (WNK‐1, WNK‐4, CUL3, and KLHL3) with different pathogenic expressiveness [1, 2, 3, 4]. Autosomal dominant variants of WNK (with no lysine [K] kinase) encode a family of kinases that increase the activity of the thiazide‐sensitive sodium‐chloride cotransporter (NCC) and increase the internalization of the renal outer medullary potassium channel. CUL3 (Cullin‐3) and KLHL3 (Kelch‐like 3) genes encode for proteins that degrade WNK. Inactivating variants of these genes increase the availability of WNK‐1 and WNK‐4 at the distal nephron level [2, 3]. Phenotypic variability of PHA II includes age at diagnosis, variable expression of hypertension, and the degree of sensitivity to thiazide diuretics [4, 5]. Pathological variants in the CUL3 and KLHL3 genes characterize a more severe phenotype [3].

In the pediatric age, PHA II also causes dehydration, muscle weakness, short stature, dental abnormalities, and developmental delay [3, 6]. In adulthood, the presentation of PHA II includes fatigue, shortness of breath, hypertension, occasionally detected hyperkalemia, hyperchloremia, and sometimes hypercalciuria [3, 7].

Very few cases of PHA II are diagnosed in hypertensive middle‐aged or elderly subjects with variable renin and aldosterone levels and different sensitivity to thiazide diuretics [8, 9, 10, 11, 12], probably owing to the rarity of the disease [7] and to a certain degree of medical inertia in excluding secondary causes of hypertension in this age.

Here, we report the case of a 55‐year‐old woman without a family history of hypertension, who had suffered from elevated blood pressure for about 20 years. Irrespective of her occasionally noted hyperkalemia, she underwent her first endocrinological consultation for elevated aldosterone levels. Following a clinical diagnosis of PHA II, genetic testing revealed two heterozygous KLHL3 mutations. To our knowledge, these variants have not been reported before and could be pathogenic. In addition, in this case, high sensitivity to hydrochlorothiazide (HTZ) was noted, and treatment was maintained at very low dosages in comparison with those reported in the literature.

## 2 Case History

**FIGURE 1:**
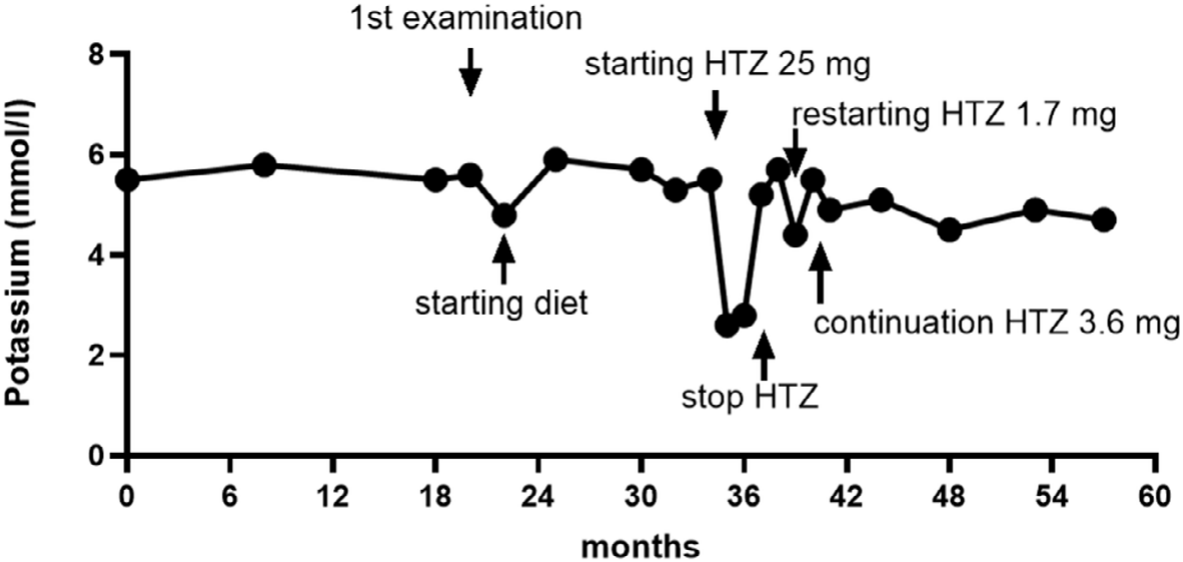
Reports on several potassium levels. Historical data before the first examination were collected. Thereafter, data are reported during low potassium diet and hydrochlorothiazide (HTZ) treatment. In brackets, the estimated HTZ posology per day is reported.

In January 2022, a hypertensive 55‐year‐old woman presented for endocrinological examination and reported a diagnostic suspicion of primary hyperaldosteronism. Her sitting aldosterone level was elevated (Table 1). The suspicion was strengthened by the aldosterone/active renin ratio (104; normal ratio < 62). This diagnostic hypothesis emerged in 2018 from examinations performed by nephrologists. Potentially interfering drugs (olmesartan medoxomil 10 mg and amlodipine 5 mg) were ongoing, and a high potassium level (Table 1) was not considered. To exclude iatrogenic effects and to evaluate aldosterone according to current guidelines [13], hypotensive treatment was changed to verapamil hydrochloride (80 mg) and doxazosin (4 mg). A 2‐month dietitian consultation was started. In May 2022, high aldosterone and potassium levels were still noted. From May 2022 to March 2023, the patient did not attend for examinations owing to fear. In April 2023, the patient again became available for further imaging and laboratory examinations. Echocardiography showed moderate left ventricular hyperplasia (10 mm; adult women's normal range 6–9 mm). Abdominal magnetic resonance showed normal adrenal morphology, and ultrasonography revealed normal blood flow and kidney morphology. Urinalyses were normal. Other forms of endocrine secondary hypertension were excluded after TSH, free‐urine cortisol, 17‐OH‐progesterone, and urine metanephrine and normetanephrine evaluation. Venous blood gas revealed low pH and bicarbonate (Table 1). Owing to the presence of hypertension, hyperkalemia, and metabolic acidosis with an estimated glomerular filtration rate (GFR) of 79 mL/min/1.73m^2^ (CPK‐EPI: CKD stage 2), a condition of PHA II was hypothesized. As the patient's only living family member (her brother) was unavailable, no family history could be gathered. After the patient's informed consent had been obtained, a sample of peripheral blood was sent to the Clinical Genetics Unit of the University of Padua (Padua, Italy) in May 2023 for genetic testing (NGS, Illumina MiniSeq, Agilent SureSelect) of genes involved in the regulation of NCC transporter activity (CUL3, KLHL3, WINK‐1, WINK‐4, SCNN1A, SCNN1G) and adrenal steroid synthesis (CYP11B1, CYP11B2). In the same period, while the results of the genetic analysis were being awaited, a course of HTZ treatment (25 mg/day) was started. The initial dosage of HTZ was chosen according to the available literature [3]. The response to treatment was very exaggerated, with a drop in potassium levels even after HTZ had been reduced to 6.25 mg/day (Figure 1). The withdrawal of HTZ resulted in recovery of the glomerular filtration rate (GFR) from 43 to 60 mL/min/1.73m^2^, but the recurrence of a high level of potassium was noted (Figure 1). Until January 2024, the patient refused to restart HTZ at the dosage of only 12.5 mg/week (about 1.7 mg/day). Thereafter, however, HTZ was restarted, and, on increasing the dosage to 25 mg/week (about 3.6 mg/day), potassium levels decreased to normal (Figure 1), GFR rose (70 mL/min/1.73m^2^), and aldosterone normalized, without full recovery of blood gas values (Table 1). In September 2024, whole‐exome sequencing was available; double heterozygous mutations of KLHL3 [RefSeq NM_017415.2: c.1594G>T p.(Arg430Trp) and RefSeq NM_017415.2: c.1288C>T p.(Val532Phe)] were found.

**TABLE 1:** Some clinical and biochemical data observed before and during endocrinological follow‐up.

	Historical data	1st endocrine examination	On low potassium diet	On HTZ 25 mg/day	Withdrawal HTZ	Restart HTZ 12.5 mg/week	HTZ 12.5 mg twice/week	Last examination
BMI (kg/m^2^)		22.2	21.8	21.3	21.7	21.3	21.8	22.6
Systolic BP (mmHg)	190	180	160	130	130	125	130	135
Diastolic BP (mmHg)	100	90	80	80	85	80	80	85
Sodium (mmol/l)	138	139	138	135	140	134	139	136
Potassium (mmol/l)	5.5	5.8	5.8	2.6	5.2	5.1	4.5	4.9
U‐sodium (mmol/24 h)	90		95			68		173
U‐potassium (mmol/24 h)	37		38			22		38
Chloride (mmol/l)			110	88	109	105		103
Calcium (mmol/l)	2.43		2.38	2.15	2.45	2.50	2.38	
Creatinine (μmol/l)	76.0		82.2	120.2	91.1	89.4	80.5	97.2
eGFR ml/min/1.73/m^2^	77		69	43	60	62	70	55
Aldosterone (ng/l)	583		867	338				195
Active renin (ng/l)	5.1		1.6	0.5				5.6
Venous blood pH			7.30	7.43	7.29	7.30	7.26	7.30
Bicarbonate (mmol/l)			22.3	31.9	23.5	23.6	26.1	27.0

*Note:* Normal range of laboratory data: sodium 136–145 mmol/L, potassium 3.5–5.5 mmol/l, U‐sodium 40–220 mmol/24 h, U‐potassium 25–125 mmol/24 h, chloride 101–109 mmol/L, calcium 2.15–2.50 mmol/L, creatinine 53.1–97.3 μmol/L, aldosterone 35–300 ng/L, active renin 5.4–34.5 ng/L, venous blood pH 7.32–7.42, and bicarbonate 24.0–28.0 mmol/L.

## 3 Outcome

At the last examination (January 2025), the patient was asymptomatic and reported normal home blood pressure (130/80 mmHg). In‐office sitting blood pressure was at a high‐normal level (135/85 mmHg). The last data are shown in Table 1. Therapies were as follows: HTZ (12.5 mg twice a week), verapamil hydrochloride (40 mg/bid), amlodipine (5 mg/day), and sodium bicarbonate (2.0 g twice a week). The patient provided written consent to use her history and data in this case report.

## 4 Discussion

PHA II is a rare disorder with genetic heterogeneity due to the different mutations involved. The prevalence of PHA II is unknown. Age at PHA II diagnosis is variable, ranging from infancy to adulthood. Inertia in the basic assessment of hypertension and the difficulty of general practitioners in accessing refined laboratory and genetic examinations could influence the diagnosis of PHA II, as in other cases of secondary hypertension. The literature reports a few families with dominant and recessive inherited PHA II and sporadic cases due to new mutations [9, 14]. Here, we report the first case of PHA II with two new heterozygous mutations of the KLHL3 [RefSeq NM_017415.2: c.1594G>T p.(Arg430Trp) and RefSeq NM_017415.2: c.1288C>T p.(Val532Phe)] gene diagnosed in middle age after a long period of hypertensive status. These variants were considered likely pathogenic, according to the Criteria of the American College of Medical Genetics.

Anecdotal cases of PHA II in adults are fairly rare. Mehandru et al. [5] described metabolic acidosis and hyperkalemia in three adult patients aged between 55 and 68 years, in whom the diagnosis of Gordon syndrome was made according to clinical evidence, without genetic evaluations, and who responded better to a strict low‐potassium diet than to salt restriction or HTZ administration. In our patient, however, a low‐potassium diet did not restore normal potassium levels. Etges et al. [10] reported a variant of the KLHL3 gene that resulted in a p.arg431trp missense mutation in a 58‐year‐old Turkish woman, in whom persistent hyperkalemia had been noted 9 years earlier, after discontinuation of a valsartan plus HTZ (12.5 mL) regimen. In this patient, L‐thyroxine was started for concomitant subclinical hypothyroidism. Zhang et al. [12] reported a case of a 54‐year‐old Chinese woman whose general practitioner diagnosed autoimmune hyperthyroidism, but a potassium level of 5.48 mmol/L went unnoticed. A homozygous missense variant of the KLHL3, c.328 A>G, gene was later identified. In our patient, thyroid function was normal. We therefore speculated that thyroid disorders and PHA II may be unrelated. Sambharia et al. [11] described two adults aged 42 and 56 years with KLHL3 variants (c1487 G>A; c1582 C>T) in whom hyperkalemia had been treated unsuccessfully with furosemide and fludrocortisone before HTZ was started.

HTZ is the most effective treatment in PHA II, but the ability of the drug to lower potassium is variable. In our case, very low dosages of HTZ (about 3.57 mg/day) were needed in order to achieve long‐term control of potassium levels, while our starting dose (25 mg/day), which was chosen according to some literature data [8, 9, 10, 15] and administered some months before genetic confirmation of the PHA II diagnosis, caused electrolyte disturbance, pH change, and dehydration within a few days.

After his initial clinical characterization of the syndrome, Gordon [1] reported treatment as a combination of a diet restricted in NA+ and K+ and the administration of thiazide diuretics, though without indicating drug dosage. In some papers, HTZ dosages were not reported [16, 17], or other therapeutic choices were made [4, 11]. The HTZ dosage of 25 mg/day has proved effective in some cases. For instance, in a 54‐year‐old woman with a missense variant of KLHL3, HTZ 25 mg/day reduced serum potassium from 5.7–5.9 to 4.87 mmol/L; after discharge from the hospital, this value remained normal (4.81–5.08 mmol/L) and blood pressure was 145/80 mmHg; this patient was, however, also on beta‐blocker and nifedipine therapy [12]. In a 17‐year‐old male with a de novo heterozygous CUL 3 mutation, HTZ 25 mg once a day (about 0.22 mg/kg bw) improved his potassium level (4.8 mmol/L) over a 3‐month follow‐up [15]. In a 58‐year‐old woman, initiation of therapy with HTZ 25 mg/day reduced potassium from 5.3 to 4.2 mmol/L, with blood pressure normalization; a temporary reduction in HTZ to 12.5 mg/day, however, led to the reappearance of hyperkalemic acidosis [10]. In two patients with a suspicion of PHA II due to WNK1 variants, HTZ was started at a dosage of 12.5 mg/day, but one needed an increase to 25 mg/day in order to normalize potassium levels [8]. In a 42‐year‐old woman, HTZ was started at 12.5 mg/day and then reduced to 12.5 mg every other day owing to hypotension, without the recurrence of hyperkalemia. In another patient, a 13‐year‐old girl, HTZ at 12.5 mg/day reduced potassium to 2.7 mmol/L, and a combination of spironolactone and HTZ was administered 5 months after diagnosis [11]. Finally, Kostakis et al. [18] reported that HTZ 0.4 mg/kg/day (about 10 mg/day) was started in a 7‐year‐old boy with a clinical diagnosis of PHA II when he was at the 5th percentile of body weight. The same patient was reassessed at the age of 20 years, when he joined the army, and the HTZ dosage was increased to 12.5 mg/day in order to maintain blood pressure and potassium levels in the normal range [18].

The data available indicate that the dosage of 12.5–25 mg/day of HTZ is most frequently administered, but that the amount of the drug must be personalized according to the clinical response. To our knowledge, very low HTZ dosages, as in our case, have not been previously employed in adults with PHA II. Sensitivity to therapy is individual and is probably linked to the gene mutation. On the other hand, a discrepancy can be observed between the normalization of potassium levels and the normalization of other clinical and biochemical data [4, 8, 12], as in our case, in which low‐dose sodium bicarbonate administration needed to be maintained, and hypotensive treatment was not interrupted. In the majority of cases reported in the literature, follow‐up was not long. So far, our patient has undergone 1 year of follow‐up on a fixed dosage of HTZ, but we do not know whether any changes in therapies will be required in the future.

## 5 Conclusion

This report underlines the fact that inertia in diagnosing secondary hypertension must be overcome. Moreover, diagnosing PHA II may also be tough in middle age when inexplicable hyperkalemia with long‐lasting hypertension and acidosis is noted. At present, the clinical diagnosis must be supported by genetic testing, which can reveal new mutations involved in the NCC function. Finally, in PHA II, the patient may be highly sensitive to HTZ, which can hypothetically be linked to altered NCC expression or thiazide pharmacodynamics due to the specific mutations, and therefore requires careful observation and follow‐up.

## Author Contributions

**Massimo Giusti:** conceptualization, data curation, writing – original draft. **Monica Repetto:** writing – original draft. **Carmela Caputo:** writing – original draft.

## Consent

Written informed consent for genetic evaluation was obtained from the patient.

## Conflicts of Interest

The authors declare no conflicts of interest.

## Data Availability

Data collected during this study are included in the case report. Additional details can be obtained from the corresponding author upon request.
